# A multi-level intervention to reduce the stigma of substance use and criminal involvement: a pilot feasibility trial protocol

**DOI:** 10.1186/s40352-023-00224-x

**Published:** 2023-05-15

**Authors:** Kelly E. Moore, Jennifer E. Johnson, Jason B. Luoma, Faye Taxman, Robert Pack, Patrick Corrigan, Jim Hart, Judge Duane Slone

**Affiliations:** 1 Department of Psychology, East Tennessee State University, 420 Rogers-Stout Hall, P.O. Box 70649, Johnson City, TN 37614 USA; 2 College of Human Medicine, Division of Public Health, Michigan State University, 200 E. 1St Street, Flint, MI 48502 USA; 3 Portland Psychotherapy Institute, 3700 N Williams Ave., Portland, OR 97227 USA; 4 Schar School of Policy and Government, George Mason University, 3351 Fairfax Drive Van Metre Hall, Arlington, VA 22201 USA; 5 College of Public Health, East Tennessee State University, 277 Lamb Hall, P.O. Box 70623, Johnson City, TN 37614 USA; 6 Department of Psychology, Llinois Institute of Technology, 3424 S State St., Chicago, IL 60616 USA; 7 Tennessee Institute for Public Service, 1610 University Avenue, Knoxville, TN 37921 USA; 8 Fourth Judicial District of Tennessee, 854 South Hwy. 92, P.O. Box 858, Dandridge, TN 37725 USA

**Keywords:** Stigma, Criminal legal system, Multi-level intervention, Staff, Drug court, Clinical trial, Protocol, Substance use disorder

## Abstract

**Background:**

Stigma associated with substance use and criminal involvement is pervasive and creates a barrier to evidence-based addiction care within the criminal legal system. Research has yet to examine a multi-level stigma intervention which targets the intersection of these stigmas among both criminal legal staff and legally-involved clients.

**Methods:**

This paper presents the protocol for a non-randomized trial of a multi-level stigma intervention called Combatting Stigma to Aid Reentry and Recovery (CSTARR) that involves two interventions: (1) training for criminal legal staff to address public stigma and (2) group-based acceptance and commitment therapy to address self-stigma among legally-involved adults enrolled in substance use treatment. Staff and client participants are engaged with a program called the Tennessee Recovery Oriented Compliance Strategy in 6 East Tennessee counties. This study examines the feasibility, acceptability, and preliminary effectiveness of CSTARR using a type 1 hybrid implementation/effectiveness trial with pre to post follow-up.

**Discussion:**

Stigma must be addressed in the criminal legal system to facilitate the uptake of evidence-based addiction care. This study is the first to evaluate a stigma intervention designed for the criminal legal setting and results will be used to inform a larger, randomized controlled trial. The rationale for this study, research design and measures, as well as potential implications for the field are described.

**Trial registration:**

This clinical trial is registered at clinicaltrials.gov with the identifier NCT05152342. Registered 11/5/2021 at https://register.clinicaltrials.gov/prs/app/action/SelectProtocol?sid=S000BIN8&selectaction=Edit&uid=U0005X4C&ts=2&cx=-u3wsbx.

Stigma has been identified as one of the most challenging barriers to substance use treatment in the criminal legal system (Avery, 2019; Wakeman & Rich, 2018). Stigmatizing attitudes about substance use, its intersecting conditions (e.g., criminal involvement), and its treatment are common in court, corrections, probation, and mandated treatment sectors. For example, staff working in the criminal legal system may think that people involved in the criminal legal system are untrustworthy, unmotivated, and unable to change, and are skeptical of addiction treatments like medications for opioid use disorder (Belenko et al., 2018; Eno, 2009; Grella et al., 2020; Mitchell et al., 2017). Stigma surrounding both substance use and criminal involvement weigh heavily on people in the criminal legal system, leading them to expect rejection and withdraw from their communities (Moore & Tangney, 2017), disengage from treatment (Conner & Rosen, 2008; Olphen et al., 2009), and experience poor health outcomes (Martin et al., 2020). Given that more than a third of people receive substance use treatment in the legal system (Smith and Strashny (n.d.)) and around 60% of legally-involved people have substance use disorders (Bronson et al., 2017), best practices for reducing the impact of stigma on people with substance use disorders in the criminal legal system are essential.

## A multi-level stigma reduction approach

Stigma is a multifaceted construct that involves several interacting levels, including structural factors (e.g., policies, funding availability) that restrict the behavior of stigmatized people and limit their access to resources, social factors that involve community members’ attitudes and behaviors toward stigmatized people, and self-factors, which involve the cognitive-affective-behavioral effects of stigma on a person (Corrigan et al., 2006; Hatzenbuehler et al., 2013; Link & Phelan, 2001). As such, researchers suggest that stigma should be intervened with at multiple levels, targeting individuals and the broader social-community environments they are in Cook et al. (2014). This has been deemed particularly important within treatment systems because stigma can prevent people from receiving effective care that is critical for their health (Nyblade et al., 2019).

Multi-level stigma interventions typically focus on two or more levels (i.e., structural, social/public, or self), and to date, have primarily focused on reducing HIV/AIDs or mental illness stigma (Rao et al., 2019). There have been very few multi-level stigma interventions addressing substance use, but existing studies suggest it is feasible to implement interventions that target multiple levels of stigma simultaneously, such as with staff and patients, within substance use treatment systems (Li et al., 2013). There have been no multi-level stigma reduction approaches tested within the context of the criminal legal system, where structural, social, and self-stigma present significant issues for substance use treatment engagement and legal compliance more broadly (Kras, 2013; Olphen et al., 2009).

## Interventions to reduce the stigma of substance use and criminal involvement

### Social/public stigma

Social stigma (also referred to as public stigma) surrounding substance use and criminal involvement is driven by a range of factors, including lack of knowledge, familiarity, and empathy, as well as harmful institutional norms (Nieweglowski et al., 2018; Nyblade et al., 2019; Rade et al., 2016). Although there are several existing interventions designed to reduce substance use stigma among healthcare and other professionals (Crapanzano et al., 2014; Livingston et al., 2012), there are considerable gaps in these trainings. First, the majority rely primarily on education-based strategies which provide information about the science of addiction, treatment, and recovery. Although educational interventions have been shown to increase knowledge, numerous studies have shown that education alone does not produce lasting changes in stigma (Corrigan et al., 2015a), and that strategies which facilitate positive interactions between non-stigmatized and stigmatized people who have lived experience (e.g., history of substance use) have longer-lasting effects (Corrigan, 2017). Second, most if not all existing stigma interventions emphasize changing stigmatizing attitudes and beliefs as the primary goal, with outcome measures reflecting this focus (Mittal et al., 2012). Decades of psychological research suggests that attitude change is not only challenging, but potentially not as meaningful as behavior change (Hayes, 2004). Despite this, stigma interventions focus almost exclusively on attitude change. Finally, to our knowledge, no existing interventions (at any level) have attempted to address the intersection of substance use with criminal involvement stigma, the latter of which has been shown to be more predictive of criminal legal staff’s negative attitudes toward addiction treatment (Moore et al., 2022). Moreover, criminal involvement stigma (e.g., believing that people who get arrested are different/bad, unable to be rehabilitated, dangerous, untrustworthy) has been well-documented among the general public, employers, criminal legal professionals, and healthcare workers, and is harmful to the health and adjustment of people involved in the legal system (Griffith et al., 2019; Martin et al., 2020; Pager et al., 2009; Pogorzelski et al., 2005). Stigma surrounding criminal involvement becomes particularly problematic when it interferes with providing evidence-based care for substance use. Specifically, beliefs that people with substance use disorders who get arrested deserve punishment rather than treatment, and expectations that people in the criminal legal system will never recover can prevent legal settings from facilitating access to high-quality addiction treatment. Taken together, these gaps suggest the need for a multi-level intervention targeting social/public stigma among criminal legal staff that addresses substance use *and* criminal involvement stigma, psychoeducation *and* contact with stigmatized people who have lived experience of addiction/incarceration, and a focus on behavioral *in addition to* attitudinal change.

### Self-stigma

Although no interventions have been developed to address self-stigma associated with criminal involvement, there have been interventions designed to address substance use self-stigma. One intervention in particular is promising: acceptance and commitment therapy (ACT) for substance use self-stigma. ACT for substance use self-stigma is a brief intervention delivered over 6 h that targets the underlying affective-behavioral mechanisms theorized to drive self-stigma and shame, including cognitive fusion with negative perceptions of oneself and one’s environment, as well as avoidance of and attempts to control painful emotional experiences (Luoma et al., 2008). Often delivered as an adjunctive component to existing substance use treatment programs, this intervention helps participants clarify their values, identify how stigma may interfere with their values, and learn strategies to persevere toward their values regardless of stigma-related thoughts and experiences. This intervention has been evaluated in a randomized controlled trial, showing improvements in treatment retention and substance use (Luoma et al., 2012). ACT for substance use self-stigma has not been implemented specifically within a criminal legal context, and also does not address the intersection of substance use and criminal involvement stigma, which may be uniquely harmful to self-worth (Gunn et al., 2018; van Olphen et al., 2009; Rutter & Barr, 2021). For example, in our team’s clinical experience, some people in mandated treatment report substance-related criminal charges (e.g., driving while intoxicated) as particularly stigmatized, whereas others view getting arrested and going to jail as more stigmatizing than substance use itself. Also, people involved in the criminal legal system often believe their criminal background is uniquely prohibitive due to required disclosure in employment and other settings (Swan, 2016). Further, although some legally-involved people may exhibit more antisocial traits and/or deflect criminal involvement self-stigma by externalizing blame (e.g., believing their addiction is the sole cause of their legal problems, believing others are to blame for their arrest), they may still experience heightened anticipated stigma and the resulting sequalae of avoidance and poor coping. These unique elements of criminal involvement stigma must addressed alongside substance use stigma in interventions being delivered with legally-involved populations.

## Stigma reduction in the criminal legal context

The criminal legal system presents unique challenges for stigma reduction. While stigma itself needs to be the target of intervention efforts, it is also an implementation issue. Broadly, criminal legal cultures are notoriously difficult to shift, which can make implementing and sustaining a new intervention, especially one that aims to reduce stigma and simultaneously increase uptake of evidence-based substance use treatment approaches, challenging (Zielinski et al., 2020). Legal system staff are generally shown to have high levels of stigmatizing attitudes about criminal involvement (Kjelsberg et al., 2007; Ware et al., 2012) and substance use disorder (Belenko et al., 2018; Grella et al., 2020), and they also have high rates of burnout, which contributes to stigma (Dir et al., 2019). Moreover, staff often have persistent negative interactions with people involved in the criminal legal system, some of which involve fear or are traumatic (Bell et al., 2019), and this may decrease their amenability to stigma reduction efforts. Because of heightened stigmatizing attitudes and potentially limited buy-in, stigma reduction may be uniquely challenging to implement in criminal legal settings. In addition, resources (e.g., time, financial, staffing) have always been limited in U.S. criminal legal agencies, making any sort of system-wide stigma training for staff a challenge, especially when the system itself spans court, detention, treatment, and community supervision sectors. Indeed, this may be why very few stigma interventions have been implemented with legal system staff thus far.

At the level of the individual involved in the criminal legal system, there are unique considerations for stigma reduction in legal settings as opposed to more traditional community-based treatment settings. People involved in the legal system may be ambivalent about engaging and developing an alliance with providers since they did not voluntarily enter treatment (Höfer et al., 2015) and may have more antisocial features or traits that increase mistrust of providers and make treatment challenging (Moore et al., 2018). The logistics of intervention delivery with legally-involved populations can also present a significant barrier, as people often lack transportation, access to money and technology, and other resources that would be necessary for engaging in treatment (Morani et al., 2011). The combination of logistical and other implementation barriers both at the client and staff levels make multi-level stigma reduction complex in the criminal legal context, and intervention approaches targeting stigma must be carefully designed to navigate these.

## A new multi-level stigma intervention

To address the aforementioned gaps, a multi-level stigma intervention for the criminal legal system, Combatting Stigma to Aid Re-entry and Recovery (CSTARR), was developed. CSTARR aims to reduce social/public stigma tied to both substance use and criminal involvement among criminal legal staff as well as the intersection of these self-stigmas among individuals with substance use disorders who are involved in the criminal legal system. The goal of CSTARR is to improve staff attitudes and behaviors toward their clients (public stigma) as well as client perspectives and engagement in SUD treatment that is mandated by the legal system (self-stigma). Both sources of stigma are thought to ultimately impact recovery and legal outcomes. CSTARR is designed to be implemented in substance use treatment mandated by the legal system, particularly in programs that involve collaborative oversight and involvement from court, detention, treatment, and probation staff, such as incarceration diversion programs. This approach allows for the delivery of two anti-stigma interventions, one designed for people in the criminal legal system receiving substance use treatment and another designed for a range of legal professionals (e.g., attorneys, probation, judges, treatment staff) who oversee those individuals as they are diverted from incarceration into treatment.

### Preliminary studies

In the year prior to the present study, CSTARR-staff was delivered at a local probation treatment agency to 20 staff members spanning probation, treatment, and administrative roles. Staff completed pre- and post-test measures and provided feedback on the intervention content and survey instrument clarity. After the training, stigmatizing attitudes about criminal involvement and perceived differences between oneself and legally-involved people decreased from pre- to post-test. Additionally, CSTARR-client was pilot tested using a virtual delivery method (i.e., Zoom videoconferencing software) with 10 individuals engaging in treatment at a drug recovery court. Preliminary results showed that this method of intervention delivery was feasible, and qualitative results showed that participants enjoyed the intervention, learned more about stigma, and gained coping skills.

## Methods

### The present study

This study uses a type 1 hybrid implementation/effectiveness design (Bernet et al., 2013) to examine the feasibility, acceptability, and preliminary effectiveness of CSTARR. The specific aims of this study are to 1) examine the feasibility, acceptability, and implementation of CSTARR-staff and CSTARR-client within an incarceration diversion program; and 2) collect pilot data on how CSTARR impacts outcomes at the client level (e.g., self-stigma, self-efficacy, coping skills), staff level (e.g., behaviors and attitudes toward stigmatized people, openness to evidence-based treatment), and program level (e.g., retention in program, substance use, disciplinary infractions). This study has been approved by the university’s Institutional Review Board and is registered on clinicaltrials.gov with the identifier NCT05152342. Recruitment/data collection with staff and client participants is ongoing. All study activities are conducted in accordance with the Declaration of Helsinki, and all participants provide consent to participate prior to engaging in the study.

### Study design overview

This study plans to recruit approximately 72 criminal legal staff and 72 individuals involved in the criminal legal system across 6 counties (i.e., 12 per county) engaged with an incarceration diversion program called the Tennessee Recovery Oriented Compliance Strategy (TN-ROCS). Interventions for staff (1 4-h session for all staff in a county) and clients (multiple group sessions with 4–6 clients per group, per county) are to be delivered over the course of 12 months, with the staff interventions occurring first, followed by the client interventions in each county. The primary goal of this pilot study is to gather data on feasibility and acceptability of the interventions with staff and clients. The secondary goal is to examine preliminary effectiveness by analyzing change in client-level and staff-level outcomes of interest across baseline, post-test, one-month, and 3-month timepoints. We also aim to evaluate data on clients’ program-level outcomes, such as continued engagement in the TN-ROCS treatment program as well as disciplinary infractions and substance use that occurs during treatment. We utilized an open trial for this study due to our main focus on feasibility; we decided against having a comparator condition because clustering of clients and staff within counties would have necessitated a larger sample size than was feasible to recruit during the study period. In addition to the study activities described above, we will hold a focus group in each county at the end of the study to discuss implementation facilitators and barriers, as well as revision of implementation strategies moving forward.

### Setting

CSTARR-staff and CSTARR-client are being delivered within the infrastructure of the TN-ROCS program, which is a drug recovery court (incarceration diversion) program operating in select counties in Tennessee. TN-ROCS was developed by Judge Duane Slone, who is a community leader and primary person facilitating stakeholder buy-in and engagement with this project. We chose this program because it allowed us to recruit multiple types of criminal legal professionals and their clients within a single program infrastructure to implement both levels of stigma interventions. TN-ROCS coordinates staff from multiple sectors (i.e., courts, detention, probation, treatment) into a unified care and judicial oversight approach for legally-involved people with substance use disorders. Upon arrest, individuals are identified as potentially eligible for TN-ROCS if substance use problems are deemed relevant to their legal involvement. A program intake is completed with a mental health treatment staff called a criminal justice liaison (CJL) during initial incarceration and a report including treatment recommendations is then reviewed by the judge who makes a final determination about acceptance into TN-ROCS. Clients accepted into the program are released and sent to an appropriate treatment setting based on their substance use needs (e.g., inpatient, residential, outpatient, medication). TN-ROCS clients appear before the judge and other team members (e.g., attorney, probation officer, treatment staff) regularly to ensure compliance with treatment. Sanctions (e.g., jail time) are used as needed to address non-compliance. Clients are typically enrolled in TN-ROCS for one year or longer.

To identify counties for inclusion in this study, Judge Slone connected us with judges in other counties, as well as the Tennessee Department of Mental Health and Substance Abuse Services (TDMHSAS), who oversee all TN-ROCS programs in the state. A list of possible Tennessee counties for this study was generated based on whether the county had the TN-ROCS program. TDMHSAS provided contact information for CJLs and other critical stakeholders in these counties who we then connected with to gauge interest in stigma reduction. Out of 10 possible counties with an operating TN-ROCS program, we chose the 6 that had the most well-established TN-ROCS program and wherein the judge reported a desire to engage with stigma reduction efforts. Five of the six counties are rural and span 3 judicial districts in Northeast Tennessee: Jefferson (rural), Grainger (rural), and Sevier (rural) counties in the 4^th^ judicial district, Monroe (rural) and McMinn (rural) counties in the 10^th^ judicial district, and Sullivan county (urban) in the 2^nd^ judicial district. Judges and CJLs were the primary stakeholders involved in the implementation planning in each county and helped create the staff- and client-level recruitment and intervention delivery plans.

### Interventions

#### Content and structure

At the staff level (CSTARR-staff), CSTARR involves a 4-h synchronous virtual training delivered via Zoom videoconferencing software, by the PI (Moore) along with a certified peer recovery specialist. The training is organized into three parts which focus on 1) psychoeducation about stigma as well as the intersection of substance use and criminal involvement, 2) contact with a peer recovery specialist, and 3) didactics around behavioral strategies (e.g., language, communication, validation) that can reduce stigma. The psychoeducation component was developed by drawing from multiple publicly available training curricula targeting substance use stigma (e.g., opioidlibrary.gov) as well as pertinent research that is used to challenge myths about addiction and criminal involvement. The contact component features a peer recovery specialist who shares personal experiences with substance use, incarceration, and stigma in Tennessee. Peer recovery specialists can have a wide range of roles, including facilitating connection to and delivery of behavioral health interventions as well as case management types of tasks; peer recovery specialists in Tennessee are certified through the Department of Mental Health and Substance Abuse Services. The behavioral strategies were drawn from literature on the use of stigmatizing language (Tran et al., 2018) as well as therapeutic strategies for interpersonal effectiveness, communication, and validation (Linehan, 1993). CSTARR-staff utilizes a mixture of visual aids (e.g., slides), media (e.g., video clips) and polls during the intervention. CSTARR-staff trainings are to be delivered for TN-ROCS staff in each county prior to the start of the CSTARR-client intervention in that county.

At the client level (CSTARR-client), CSTARR involves ACT for substance use self-stigma (Luoma et al., 2008), delivered virtually or in-person, that our team adapted to address the intersection of substance use and criminal involvement stigma. ACT for substance use self-stigma involves 6 h of group-based therapy sessions delivered by the PI (Moore) and a doctoral student in clinical psychology virtually via Zoom or in-person, depending on rurality, the capabilities of each county, and Covid-19 precautions. The content teaches values clarification, psychoeducation on self-stigma, and skills for reducing avoidance of stigma stressors. The underlying theory of ACT emphasizes the acceptance of thoughts and other negative internal experiences rather than changing these experiences, but several skills for regulating difficult emotions are incorporated in our version of the intervention. The intervention involves didactic and experiential exercises as well as client discussion. The CSTARR-client intervention is to be delivered after CSTARR-staff training has occurred in each county.

Staff and client participants are not prohibited from engaging with other trainings or treatments throughout the course of the study. Clients are expected to engage in ongoing substance use treatment that is part of the TN-ROCS treatment plan, and staff may be required to complete other mandatory professional trainings.

#### Therapist training

The PI has experience delivering didactic trainings to criminal legal staff audiences and will plan to consult as needed to ensure appropriate delivery and receive feedback on content and structure. The PI and graduate student delivering the client interventions have experience delivering behavioral interventions as well as specific training in acceptance and commitment therapy. The PI is a licensed clinical psychologist and the trainee has two years of clinical experience. The PI and trainee will engage in regular consultation with the intervention developer (Luoma) who will review video recordings of the training to provide feedback on clinical skills and content delivery. Treatment fidelity scales and interventionist training procedures will be developed throughout the study to facilitate fidelity rating of therapists.

### Participants and procedures

#### CSTARR-staff

The CSTARR-staff intervention is scheduled according to each county’s court calendar to facilitate TN-ROCS staff availability. To recruit our target sample of 72 staff across 6 counties, the judge in each county will distribute an advertisement for the CSTARR-staff training that includes a link to register. Staff will be strongly encouraged to participate but not required to. When staff register, they will automatically be sent the training details (via Zoom) and then emailed an invitation to complete the online baseline survey which includes the informed consent. Staff are permitted to register and attend the training without completing the baseline survey. Any staff affiliated with the TN-ROCS program who have interacted (either virtually or in- person) with a TN-ROCS client in the past 90 days are eligible to participate. Staff are asked to complete an anonymous feedback survey during the last 10 min of the training and are sent a link to complete the post-test survey at that time. Staff are automatically emailed with the one-month and three-month follow-up survey links. For completing the pre- and post-test surveys, staff receive a $5 e-gift card. For the follow-up surveys, they are emailed a $10 gift card. We anticipate retaining 75% of staff by the 3-month follow up. The training is audio-recorded. We will continue to approach staff for follow-up regardless of missing data at a prior timepoint. Email and phone call reminders will be used to increase completion of online surveys.

#### CSTARR-client

To recruit our target sample of 72 TN-ROCS clients across 6 counties (i.e., approximately 12 per county), each county’s CJL will distribute a study advertisement when doing treatment intakes with new clients. CJLs (and judges) will strongly encourage clients to consider participating but it will not be required as part of their involvement in TN-ROCS. Interested clients sign a recruitment waiver so their contact details can be released to the research team. The Project Coordinator then contacts clients via phone to have an in-depth discussion of procedures, risks, benefits, and human subjects protections as part of the informed consent process. Consented clients then proceed to complete their baseline interview over the phone. Any TN-ROCS clients (i.e., arrested, have evidence of substance use problems, are accepted into the TN-ROCS program), will be eligible except those deemed by the CJL to have debilitating mental illness or violence risk. There are no exclusion criteria around stigma (e.g., those reporting low self-stigma) in order to gauge CSTARR utility for all clients. At the end of each intervention session, clients are asked to complete an anonymous online feedback form, and their post-test survey (completed over the phone with a research assistant) is scheduled to take place immediately after the last intervention session. Participants receive a $5 giftcard for the baseline and post-test interviews, and a $10 gift card for the 1- and 3-month follow up assessments. To offset the time and logistical burden of completing the intervention, participants will receive up to $30 in gift cards if they attend the entire intervention. All group sessions are audio-recorded. We anticipate retaining 75% of clients across the 3 sessions and 50% by the 3-month follow-up. We continue to approach clients for follow-up regardless of missing data at a prior timepoint. Phone calls, texts, and/or email reminders are used to increase completion of surveys.

#### Focus group

A 1.5 h focus group with approximately 10 key stakeholders (e.g., judges, CJLs, administrators) who participated in the CSTARR-staff training will occur at the end of the study. The focus group will be led using a semi-structured question guide aimed at understanding 1) feedback on intervention delivery 2) discussion of barriers, 3) suggestions for improvement, and 4) ideas for sustainment. Informed consent will involve explaining focus group procedures, risks/benefits, and incentives for participation ($20 giftcard). The group will be audio-recorded to facilitate transcription. Data will be used to understand barriers and facilitators of implementation to inform future study planning.

### Staff assessments

Demographic data on staff (i.e., age, sex, race, education level, agency, position, job tenure, interpersonal contact with people who have substance use and/or criminal backgrounds, and organizational stress) is gathered as part of the baseline assessment.

#### Primary staff outcomes

We will examine change from the baseline to the three-month follow-up timepoint in the following measures:

- Perspectives on Stigma Reduction. The Perspectives on Stigma Reduction scale (PSR) is a 24-item internally developed measure that assesses perspectives on stigma (e.g., importance, impact on people involved in the criminal legal system) and stigma reduction strategies (e.g., openness to learning new strategies, use of nonjudgmental behaviors), among criminal legal professionals. 20 of the items are rated on Likert scales ranging from 1 (strongly disagree) to 9 (strongly agree), and 4 items are rated on a scale from 1 (0% of the time) to 9 (100% of the time). Items with the same response scales are summed to create total composite scores. Higher scores indicate more favorable views about stigma reduction.
- Difference and Disdain. The Difference and Disdain scale (DaD) (Corrigan et al., 2015b) is a 9-item measure that assesses how individuals view others in stigmatized groups compared to the general population. Responses are rated on a scale from 1 to 9, where 1 represents a belief that stigmatized people are similar to/favorable compared to others in the general population and 9 represents a belief that they are different from/unfavorable compared to the general population. We adapted this measure to ask about people with substance use problems and then criminal involvement (18 items total). Each scale ranges from 9–81 with higher scores indicating more differences/disdain. This measure has been shown to be valid for assessing public stigma in multiple studies (Corrigan, 2017; Corrigan et al., 2021).
- Social Distance. We use 10 items to assess desired social distance from people with a criminal background (separately asking about violent and nonviolent criminal histories) and 5 parallel items to assess desired social distance from people with histories of addiction. Items were adapted from the Bogardus social distance measure (Bogardus, 1933) to capture how staff would feel about interacting with (e.g., working, living next to, talking to, marrying) people with these backgrounds. Responses are rated on a Likert scale ranging from very uncomfortable to very comfortable, with the total score ranging from 10 to 50. Higher scores indicate less desired social distance. Variations of the Bogardus social distance items have been used widely and demonstrate good reliability and validity among various samples (Compton et al., 2006; Geisinger, 2010).
- Stigmatizing Attitudes Toward Criminal Involvement. The Attitudes Toward Prisoners scale (ATP) (Melvin et al., 1985) is a 36-item measure that assesses attitudes, beliefs, and negative stereotypes about “prisoners,” with responses rated on 5 point Likert scale from disagree strongly to agree strongly. We changed the word prisoners to “justice-involved people” throughout the scale to remove stigmatizing language but also because the staff we are targeting primarily work with people who are not currently incarcerated. Negatively worded statements are reverse coded for a summed total score ranging from 36 to 180. Higher scores indicate more positive attitudes toward people involved in the criminal legal system. The ATP has been used widely with criminal legal staff samples and demonstrates good internal consistency and validity (Kjelsberg et al., 2007; Ware et al., 2012).
- Beliefs About Addiction. The Addiction Beliefs Inventory (ABI) (Luke et al., 2002) is a 30-item measure containing 8 subscales that assess how people think about substance use, including beliefs about controllability, the disease model of addiction and genetic susceptibility, the need for professional treatment, how responsible someone with substance use is for their actions and recovery, self-medication, and beliefs about addiction being a moral weakness. Responses are rated on a 5-point Likert scale ranging from strongly disagree to strongly agree, with higher scores representing more agreement with the beliefs represented in the subscale. The ABI is often analyzed as subscales rather than a total score and demonstrates acceptable reliability and validity in multiple studies (Jordan et al., 2002; Luke et al., 2002).
- Opinions about Medication for Opioid Use. An adapted version of the Opinions about Medication-Assisted Treatment scale (aOAMAT) (Friedmann et al., 2016) will be used to evaluate how staff think about the treatment of OUD with methadone and buprenorphine among legally-involved populations. Responses are rated on a 6-point Likert scale (1 = strongly disagree, 5 = strongly agree, and we added a sixth response option if they had never heard of the medications). Methadone and buprenorphine were included in the same item to reduce the measure’s length, and naltrexone was not included (e.g., “Methadone and/or buprenorphine should be available as a lifelong treatment option”). This collapsed the 18 original items into 9; 2 additional items were added regarding the administration of medications during incarceration. This adapted measure has been shown to be reliable and valid with criminal legal staff (Moore et al., 2022).

#### Secondary staff outcomes

We will examine change from the baseline to the 3-month follow-up timepoint in the Dual-Relationship Inventory-Short Form (DRI-SF) (Gochyyev & Skeem, 2019), a 9-item measure adapted from the DRI and DRI-revised (Skeem et al., 2007) that was originally designed to be used in parallel forms with probation officers and their clients. The DRI assesses qualities of the relationship and working alliance between probation officers and clients. Responses are rated on a scale ranging from 1 (never) to 7 (always), with total scores ranging from 9 to 63. Higher scores indicate better relationships with clients. The DRI-Revised has been shown to be valid and internally consistent in a sample of probation officers and clients (Skeem et al., 2007). The DRI-SF has psychometrically sound properties comparable to the original DRI-R and has been validated in samples of probation officers and treatment staff (Gochyyev & Skeem, 2019).

#### Staff implementation outcomes

Feasibility will be assessed by examining intervention recruitment and retention rates (i.e., including people who leave the intervention prematurely) as well as survey attrition. We will use two measures to assess implementation outcomes. The first is a set of 12 internally developed items that are administered in an anonymous survey after the CSTARR-staff training. Items ask about comfort level with the material, how useful and relevant the training was, how likely staff are to use stigma reduction strategies, and how likely staff would be to recommend the training. Responses are rated on Likert scales ranging from 1 to 5, with total scores ranging from 12 to 60 and higher scores indicating more acceptability, and there are open-ended questions asking about suggestions for improvement. We will also use a 12-item measure that captures whether staff value CSTARR and how they feel about its implementation after the training.

### Client assessments

To characterize the client sample, we will gather data on sociodemographics (e.g., age, sex, race, education level, employment and housing situation) as well as substance use severity, criminal behavior/arrests, and substance use treatment experience as part of the baseline assessment.

#### Primary client outcomes

We will examine change from the baseline to the 3-month follow-up timepoint in the following measures:

- Self-stigma. We will use the Substance Abuse Self-Stigma Scale (SASS) (Luoma et al., 2013) to assess self-stigma associated with substance use and criminal involvement. The SASS is a 41-item measure that assesses self-devaluation, fear of experiencing stigma, avoidance of stigma, and values disengagement among people with substance use problems. Responses are rated on a scale from 1 (never or almost never) to 5 (very often), with total scores ranging from 41 to 205 and higher scores indicating more self-stigma. We adapted this measure to ask about both criminal involvement and substance use stigma within the items in this measure (e.g., “People think I’m worthless if they know about my substance use or criminal history”). The SASS scale has been shown to be reliable and valid for assessing self-stigma among people with substance use problems (Brown et al., 2015; Luoma et al., 2013).
- Shame. The Internalized Shame Scale (ISS) (Cook (n.d.)) is a 24-item measure that includes shame-related thoughts and experiences, which are considered central to self-stigma. Responses are rated from 0 to 4, where 0 is never and 4 is almost always, with the total score ranging from 40–120 and higher scores indicating more internalized shame. The ISS has been used to track changes in shame across clinical interventions, including ACT for substance use self-stigma (Luoma et al., 2013) and is shown to be reliable and valid in a variety of clinical and non-clinical samples (Goffnett et al., 2020; Rybak & Brown, 1996).
- Self-efficacy. We use a 1-item internally developed measure to assess the degree to which clients feel they can cope with unfair judgments from the community regarding their substance use or criminal history. The item is rated on a scale from 1 to 10, with 1 being lowest expectation of success and 10 being the highest expectation of success.
- Coping skills. We use 28 items from the Dialectical Behavior Therapy Ways of Coping Checklist (DBT-WCCL) (Neacsiu et al., 2010) to assess client use of specific skills being taught in the CSTARR-client intervention. The DBT-WCCL is originally a 59-item measure that captures client use of adaptive and maladaptive coping skills and is widely used among people attending DBT skills interventions, which is another third-wave behavioral therapy similar to ACT. This measure has been used to assess improvement across treatment among people with various degrees/severity of mental illness (Brown et al., 2019; Kells et al., 2020; Stein et al., 2016). We utilize items that capture skills being taught in CSTARR-client (e.g., engaging in valued actions, acceptance of thoughts and feelings, emotion regulation techniques such as relaxation) as well as maladaptive coping skills we believe CSTARR-client may reduce, such as externalization of blame, avoidance, and social withdrawal/isolation.

#### Secondary client outcomes

We ask clients whether or not they are still enrolled in the TN-ROCS program at the 1- and 3-month follow-up timepoints and the types of treatment they are engaged in (e.g., outpatient, medications). We also assess for instances of substance use, criminal involvement leading to arrest, positive urine drug screens, and disciplinary infractions that have occurred during treatment. If we are unable to contact participants at follow-up, we ask CJLs whether clients are still currently enrolled in the TN-ROCS program and if not, record the reason for dropout.

#### Client implementation outcomes

Feasibility is assessed by examining intervention recruitment, attendance, and retention rates as well as survey attrition. We assess acceptability of the client intervention using 12 internally developed items that are administered in an anonymous survey after each treatment session that clients attend. Items ask about comfort level with the material and how useful and relevant the group content is. Responses are rated on Likert scales ranging from 1 to 5, with total scores ranging from 12 to 60 and higher scores indicating more acceptability. There are also open-ended questions asking about suggestions for improvement.

### Data analysis plan

#### Preliminary analyses

All data will be entered directly into the Redcap survey platform (directly by staff completing the online survey, via research staff manual entry during phone interviews with clients). Data will be exported, cleaned, and managed using Statistical Package for the Social Sciences (SPSS). Data from all counties will be pooled for staff and client assessments. We will run descriptive statistics (i.e., ranges, means, frequencies) and transform variables to achieve normality as needed.

#### Implementation

Feasibility will be analyzed by calculating client and staff consent and refusal rates, attendance, participation (i.e., in-group discussion), pre- and post-survey completion, and follow-up attrition, as well as review of intervention delivery technical issues. To understand the acceptability of staff and client intervention content, we will aggregate anonymous feedback data. Qualitative data from staff focus groups will be aggregated, coded, and interpreted to better understand implementation barriers and facilitators using a top-down content analysis approach (Hsieh and Shannon, 2005) in Nvivo software.

#### Effectiveness

Pre- to post- change in staff and client assessments will be analyzed using repeated measures factorial ANOVAs that compare pre, post-, 1-month, and 3-month follow-up data for clients and staff. Tukey’s posthoc comparisons will be used to pinpoint significant mean differences between timepoints. We will examine main and interaction effects to test whether mean differences in staff assessments depend on type of profession (assuming adequate sample sizes). Omega squared effect size estimates will be computed. We will control for relevant staff- and client-level variables. Additional analyses may include testing whether client and staff change scores (3-month survey score subtracted from pre-test) predict TN-ROCS program-level outcomes such as retention and disciplinary infractions. Significant effects will be designated by *p* < 0.10 and meaningful effect size estimates.

#### Data protection and monitoring

All research ethics and HIPAA protocols will be followed during the trial to protect the confidentiality of client and staff data. Any significant changes to the trial protocol will be communicated to the institution’s IRB and approved prior to implementing. Such changes will also be reflected in the clinicaltrials.gov registry. Adverse events are queried at each client baseline and follow-up interview; any that occur during the trial will be reported to the IRB. After the data is analyzed, results will be communicated to the sponsor and used to finalize the CSTARR treatment manual containing the staff and client interventions.

## Discussion

Stigma creates significant barriers to receiving addiction care within the legal system and must be addressed in order to increase the uptake of evidence-based programs and ultimately improve legal and recovery outcomes for people involved in the criminal legal system. However, there are no interventions currently targeting stigma tied to substance use and criminal involvement among staff or clients within the criminal legal system, which is a uniquely complex and challenging implementation setting for stigma reduction. Our study begins to address this major gap by providing pilot data on a promising set of interventions for legal system staff and clients that address stigma tied to addiction and criminal involvement. Our interventions are well-grounded in the science of stigma reduction and our team has the relevant expertise in stigma, addiction, and criminal legal intervention implementation to accomplish this project.

The primary goal of this study is to determine how feasible it is to implement a suite of interventions in a multi-level framework within the criminal legal system, both from staff and client perspectives, and to gauge how acceptable the intervention content is to staff and clients. Through gathering staff and client anonymous feedback on our intervention content, we will identify which aspects of the intervention are most meaningful and impactful, which aspects need further refining, and whether the structure and delivery of the interventions work well (e.g., length of training, virtual format). For example, this study will be the first to demonstrate the value of involving a person with lived experience of addiction and incarceration in a stigma reduction training for criminal legal staff. Moreover, this study will be the first to demonstrate the utility of a client self-stigma intervention targeting both addiction and criminal involvement. We hope that the results of this study will indicate which elements of the staff training “stick” to improve attitudes and behaviors toward legally-involved people with SUDs, and which aspects of the self-stigma intervention are most helpful for clients as they move throughout the TN-ROCS program. Additionally, we hope to observe improvements in staff stigmatizing attitudes and willingness to engage in behaviors that are more supportive (vs. punitive and stigmatizing) toward the clients they work with, as well as improvements in client self-efficacy, coping skills, and self-stigma. One of the primary deliverables of this project will be a finalized manual and set of survey instruments to be examined as part of a more rigorous randomized controlled trial.

Through stigma reduction at the social/public and self levels within the criminal legal system, we hope to impact the culture to be more supportive of addiction treatment. In addition, we hope this study begins to bring awareness to the pervasive and damaging structural stigma surrounding addiction and criminal involvement. For example, even with improved acceptability of addiction treatment by staff and better engagement in treatment among clients, the criminal legal culture cannot change without broader acceptance of people with criminal/addiction histories in our society. This is necessary to facilitate better allocation of funding to research these issues and better treatment of these individuals within our healthcare and other systems. The criminal legal context is a starting point where stigma reduction has the potential to be impactful, but it is the tip of the iceberg.

### Potential limitations

Although this is the only multi-level stigma intervention being developed and tested thus far within the criminal legal system, there are limitations to our study design. Because we are working with staff and clients within a single program (i.e., TN-ROCS) in a single region of the U.S., we are limited in what we can generalize to other geographic areas or drug recovery programs. In particular, the region this study is being implemented in is largely composed of people identifying as non-Hispanic white, which means that future studies that include people of color will be needed to refine intervention content and ensure its generalizability to more diverse groups. Moreover, all clients taking part in TN-ROCS were mandated, and this study’s results may not generalize to samples of legally-involved people entering substance use treatment voluntarily; potential differences in the implementation and impact of CSTARR with legally-involved clients voluntarily enrolled in treatment should be examined in future research. In addition, we make an assumption that clients and staff interact with each other during this study, but we do not gather direct evidence of this. This is an eligibility criterion for staff (must have had interacted with TN-ROCS clients in the past 90 days), and the structure of the program requires that clients appear before the judge as well as other members of the TN-ROCS team on a regular basis to monitor their progress in the program. However, we will not be able to ensure whether or not the clients we are delivering the interventions to have interacted with the staff that participated in the staff trainings, and vice versa. This limits our ability to draw conclusions about the trickle-down impacts of social/public stigma reduction on client-level outcomes. Further, not having a control group limits our ability to draw conclusions about this treatment’s effectiveness, and because our multi-level intervention is being implemented within the context of another set of interventions (i.e., TN-ROCS provides a range of substance use treatments), it is possible that our outcomes will be attributed to TN-ROCS rather than the stigma interventions. Finally, our outcome measures are limited in the sense that they are all self-reported, which is not ideal for assessing things like urine drug analysis results. Also, due to the dearth of research on criminal involvement stigma, our stigma measures were largely validated for substance use stigma only. The psychometric properties of all adapted measures will be examined as part of the data analysis process. These are important factors we will take into consideration in a more rigorous randomized controlled trial of this intervention. Additionally, we anticipate a great deal of heterogeneity in client-level intervention implementation across counties due to differences in size of the TN-ROCS program, court schedules, logistics, and support from CJLs. Our ability to combine pre-, post- and follow-up data across counties to gauge the preliminary effectiveness of the client-level intervention will be limited. Finally, we expect to encounter a variety of logistical challenges with the virtual delivery of interventions that will speak to the feasibility of our design but also inform the methods of a larger, controlled trial.

## Conclusions

There is a need for stigma reduction efforts tailored for the criminal legal context to increase substance use treatment access and engagement. This study begins to address this gap and has implications for the broader acceptance of addiction treatment within the criminal legal system, as well as health equity and legal/recovery outcomes for legally-involved people.

## Data Availability

Not applicable.

## Abbreviations

CSTARR: Combatting Stigma to Aid Reentry and Recovery
TN-ROCS: Tennessee Recovery Oriented Compliance Strategy
CJL: Criminal Justice Liaison
ACT: Acceptance and Commitment Therapy
SPSS: Statistical Package for the Social Sciences
